# Association between exercise and health-related quality of life and medical resource use in elderly people with diabetes: a cross-sectional population-based study

**DOI:** 10.1186/s12877-020-01750-1

**Published:** 2020-09-07

**Authors:** Chien-Cheng Huang, Chien-Chin Hsu, Chong-Chi Chiu, Hung-Jung Lin, Jhi-Joung Wang, Shih-Feng Weng

**Affiliations:** 1 Department of Emergency Medicine, Chi Mei Medical Center, Tainan, Taiwan; 2 Department of Senior Services, Southern Taiwan University of Science and Technology, Tainan, Taiwan; 3 Department of Environmental and Occupational Health, College of Medicine, National Cheng Kung University, Tainan, Taiwan; 4 Department of Biotechnology, Southern Taiwan University of Science and Technology, Tainan, Taiwan; 5 Department of General Surgery, E-Da Cancer Hospital, I-Shou University, Kaohsiung, Taiwan; 6 Department of Emergency Medicine, Taipei Medical University, Taipei, Taiwan; 7 Department of Medical Research, Chi Mei Medical Center, Tainan, Taiwan; 8 Allied AI Biomed Center, Southern Taiwan University of Science and Technology, Tainan, Taiwan; 9 Department of Healthcare Administration and Medical Informatics, College of Health Sciences, Kaohsiung Medical University, 100 Shin-Chuan 1st Road, Kaohsiung, 807 Taiwan; 10 Department of Medical Research, Kaohsiung Medical University Hospital, Kaohsiung, Taiwan; 11 Center for Big Data Research, Kaohsiung Medical University, Kaohsiung, Taiwan; 12 Center for Medical Informatics and Statistics, Office of R&D, Kaohsiung Medical University, Kaohsiung, Taiwan

**Keywords:** Diabetes, Elderly, Exercise, Medical resource use, Quality of life

## Abstract

**Background:**

Exercise improves glycemic control and functional capacity in elderly people with diabetes; however, its effect on health-related quality of life (HRQoL) and medical resource use remains unclear. This study aims to clarify the effect of exercise.

**Methods:**

Using the data from National Health and Nutrition Examination Survey between 2007 and 2016, we identified 1572 elderly people with diabetes for this cross-sectional population-based study. Demographic characteristics, health conditions, comorbidities, HRQoL, and medical resource were compared among four groups (no exercise, low-intensity exercise, moderate-intensity exercise, and high-intensity exercise).

**Results:**

The mean age of all participants was between 71.5 and 73.3 years. Male participants with higher education performed more exercise than their counterparts. The moderate- and high-intensity groups reported better general health condition than the no exercise group. Depression and worse health were more common in the no exercise group. Participants in the moderate-intensity exercise group had lower risk for depression than those in the no exercise group (adjusted odds ratio: 0.13, 95% confidence interval: 0.02–0.92) after adjusting for demographic characteristics, health conditions, and comorbidities, whereas participants in the low- and high-intensity exercise did not have a lower risk. The no exercise group had the highest proportions of emergency, hospitalization, and total healthcare visits.

**Conclusions:**

Exercise is associated with better HRQoL, and lack of exercise is associated with higher medical resource use in elderly people with diabetes. Encouraging exercise is recommended in this population.

## Background

The world’s population is aging, which affects all fields of societies, including health care system, medical expenditure, labor, finance, demands for goods and services, and family structures [1]. In the United States, elderly population (aged ≥65 years) was 13.7% of the total population in 2012 and projected to be 16.8% in 2020 and 20.3% in 2030 [2]. The prevalence of type 2 diabetes is higher in elderly people. In 2011, > 25% of elderly people have diabetes [3]. Elderly people with diabetes will have substantial microvascular and cardiovascular complications as well as increased risks for frailty, institutionalization, and mortality [3].

Different forms of exercise, including aerobic, resistance, and flexibility, are the cornerstone of diabetic management, especially in elderly people with functional decline [4, 5]. Exercise improves glycemic control, cardiovascular function, muscle strength, and functional capacity in elderly people with diabetes [4, 5]. Exercise is also proved to improve frailty, which is a syndrome associated with increased risks of morbidity and mortality and has higher prevalence in elderly people with diabetes than in those without [4, 5]. Health-related quality of life (HRQoL) and medical resource use are important for elderly health care [6]. Many studies have reported the benefits of exercise for elderly people with diabetes, but the effects on health-related quality of life HRQoL and medical resource use remain unclear. Therefore, we conducted this study to compare HRQoL and medical resource use between elderly people with diabetes with and without exercise.

## Methods

### Data sources

The National Health and Nutrition Examination Survey (NHANES), a program by the National Center for Health Statistics of the Centers for Disease Control and Prevention [7], contains interviews and physical examinations and aims to assess the health and nutritional status of adults and children in the United States [7]. Given the dramatic increase in elderly population in the United States, NHANES play an important role with public health agencies to increase the knowledge of the health status of this population [7]. The interviews were conducted one person at a time at the participants’ homes [7]. The study team consisted of a physician, medical and health technicians, and dietary and health interviewers [7]. The interviewers used an advanced computer system to enter data in real time [7]. The average length of an interview was 2–3 h, but examinations varied, depending on the age of the participants [7].

### Study design, setting, and participants

The estimated population proportion for the target outcome is 20%, and the number of elderly people with diabetes is 12,000,000 in the United States [8]. After calculation, the minimum number of necessary samples is 246 based on the 95% confidence interval (CI) that the real value is within ±5% of the measured/surveyed value. Using the data on diabetes from the NHANES between 2007 and 2016, we identified 1572 elderly participants for this cross-sectional population-based study. Demographic characteristics (sex, age, race, education, marital status, and family poverty index ratio), health conditions (general health condition, body mass index, cigarette smoking, and alcohol drinking), comorbidities (hypertension, hypercholesterolemia, coronary heart disease, chest pain, sleep disorders, and malignancy), HRQoL (depression, worse health compared with the past year, fever in the past year, asthma attack in the past year, and visits to mental health professionals in the past year), and medical resource use (routine place to go for healthcare, doctor’s office or health maintenance organization as most often place for healthcare, clinic or health center as most often place for healthcare, emergency department [ED] as most often place for healthcare, number of healthcare visits/year, hospitalized overnight in the past year, ED visit for asthma in the past year) were included for the analyses.

### Definitions of variables and outcomes

The participants were categorized into following groups: (1) no exercise (*n* = 1119): no habit of exercise; (2) low-intensity exercise (*n* = 195): having a positive habit of exercise but have not reached the level of moderate-intensity exercise; (3) moderate-intensity exercise (*n* = 118): at least 150 min of moderate aerobic activity (e.g., cycling or walking) or 75 min of vigorous aerobic activity (e.g., running or playing basketball) every week; and (4) high-intensity exercise: at least 300 min of moderate aerobic activity or 150 min of vigorous aerobic activity every week. Family poverty index ratio was defined as the ratio of family income to poverty [9].

### Ethics statement

The NHANES is a publicly available database and approved by the National Center for Health Statistics institutional review board. All participants provided written informed consents for their participation in the NHANES. The current study was also approved by the Institutional Review Board of Kaohsiung Medical University (IRB number: KMUHIRB-EXEMPT(I)-20,190,033).

### Statistical analysis

We used χ^2^ test for categorical variables and analysis of variance for continuous variables to compare demographic characteristics, health conditions, comorbidities, HRQoL, and medical resource use among the four groups. Multivariate logistic regressions were used to compare HRQoL and medical resource use among the four groups by adjusting for sex, age, race, education, marital status, general health condition, family poverty index ratio, body mass index, cigarette smoking, alcohol drinking, hypertension, cholesterol, chronic heart disease, chest pain, sleep disorders, and cancer. SAS 9.4 (SAS Institute Inc., Cary, NC, USA) was used for data analysis. Significance level was set at *p* < 0.05 (two-tailed).

## Results

The male proportion was lowest in the no exercise group (49.7%) and highest in the high-intensity exercise group (72.1%, Table 1). The mean ± standard deviation ages were 73.3 ± 5.2 years, 72.4 ± 4.8 years, 72.5 ± 5.2 years, and 71.5 ± 5.0 years in the no exercise, low-intensity exercise, moderate-intensity exercise, and high-intensity exercise groups, respectively. The non-Hispanic white race was predominant among the four groups. The number of participants who had high school education or less was the highest in the no exercise group (48.1%), whereas the number of those with college degree or higher was the highest in the other three groups. Analyses for marital status showed that the number of married participants was the highest among all groups. Self-report of general health condition as “Excellent/Very good/good” was lowest in the no exercise group (44.3%) and highest in the moderate-intensity (73.7%) and high-intensity exercise (69.3%) groups. The number of participants who had a body mass index of ≥25.0 kg/m^2^ and smoked cigarettes were the highest in the no exercise group. The high-intensity exercise group had the highest number of alcohol drinkers (70.7%). The no exercise group had the highest number of participants diagnosed with hypertension (76.9%) and chest pain (31.3%). The low-intensity exercise group had the highest number of patients with hypercholesterolemia (69.2%) and malignancy (25.6%). The moderate-intensity exercise group had the highest number of participants with coronary heart diseases (17.8%), and the high-intensity exercise group had the highest proportion of individuals with sleep disorders (38.6%).

**Table 1:** Comparison of demographic characteristics, health conditions, and comorbidities among the four groups by descriptive analyses

Variables	No exercise n = 1119	Low-intensity exercise *n* = 195	Moderate-intensity exercise *n* = 118	High-intensity exercise *n* = 140	*p*-value
Demographic characteristic					
Sex					
Male	556 (49.7)	88 (45.1)	74 (62.7)	101 (72.1)	< 0.001
Female	563 (50.3)	107 (54.9)	44 (37.3)	39 (27.9)	
Age	73.3 ± 5.2	72.4 ± 4.8	72.5 ± 5.2	71.5 ± 5.0	< 0.001
Race					
Non-Hispanic white	471 (42.1)	76 (39.0)	58 (49.2)	48 (34.3)	< 0.001
Non-Hispanic black	282 (25.2)	61 (31.3)	24 (20.3)	26 (18.6)	
Hispanic (Mexican American or other)	284 (25.4)	32 (16.4)	25 (21.2)	40 (28.6)	
Other (including multiracial)	82 (7.3)	26 (13.3)	11 (9.3)	26 (18.6)	
Education					
< High school	538 (48.1)	50 (25.6)	35 (29.7)	37 (26.4)	< 0.001
High school or equivalent	249 (22.3)	46 (23.6)	26 (22.0)	25 (17.9)	
College graduate or above	332 (29.7)	99 (50.8)	57 (48.3)	78 (55.7)	
Marital Status					
Married	600 (53.6)	94 (48.2)	70 (59.3)	77 (55.0)	0.009
Never married	36 (3.2)	11 (5.6)	3 (2.5)	11 (7.9)	
Widowed	319 (28.5)	60 (30.8)	28 (23.7)	23 (16.4)	
Living with partner, separated, or divorced	164 (14.7)	30 (15.4)	17 (14.4)	29 (20.7)	
Family poverty index ratio	2.1 ± 1.3	2.5 ± 1.4	2.8 ± 1.4	2.5 ± 1.5	< 0.001
Health Condition					
General health condition					
Excellent/Very good/good	496 (44.3)	126 (64.6)	81 (73.7)	97 (69.3)	< 0.001
Fair/Poor	623 (55.7)	69 (35.4)	31 (26.3)	43 (30.7)	
Body mass index, kg/m^2^					
<18.5	9 (0.8)	0 (0.0)	0 (0.0)	1 (0.7)	0.107
18.5–25.0	165 (14.8)	43 (22.1)	19 (16.1)	28 (20.0)	
≥ 25.0	945 (84.5)	152 (78.0)	99 (83.9)	111 (79.3)	
Cigarette smoking	113 (10.1)	13 (6.7)	7 (5.9)	99 (6.4)	0.144
Alcohol drinking	589 (52.6)	102 (52.3)	77 (65.3)	99 (70.7)	< 0.001
Comorbidity					
Hypertension	861 (76.9)	147 (75.4)	86 (72.9)	96 (68.6)	0.149
Hypercholesterolemia	693 (61.9)	135 (69.2)	71 (60.2)	90 (64.3)	0.232
Coronary heart disease	193 (17.3)	23 (11.8)	21 (17.8)	19 (13.6)	0.204
Chest pain	350 (31.3)	55 (28.2)	27 (22.9)	34 (24.3)	0.105
Sleep disorders	365 (32.6)	70 (36.9)	31 (26.3)	54 (38.6)	0.159
Malignancy	259 (23.2)	50 (25.6)	29 (24.6)	31 (22.1)	0.851

Data are expressed as n (%) or mean ± standard deviation

When comparing HRQoL, the no exercise group had the highest number of participants who were depressed over the last 2 weeks (10.7%) and who had worse health compared with the past year (17.9%, Table 2). The high-intensity exercise group had the highest number of participants who had fever (11.4%), asthma attack (6.4%), and visits to mental health professionals (6.4%) in the past year; however, the differences compared with other groups were not significant.

**Table 2:** Comparison of HRQoL among the four groups by descriptive analyses

Subtype of HRQoL	No exercise n = 1119	Low-intensity exercise n = 195	Moderate-intensity exercise n = 118	High-intensity exercise *n* = 140	*p*-value
Depression over the last two weeks					
Yes	120 (10.7)	16 (8.2)	1 (0.9)	8 (5.7)	0.002
No	999 (89.3)	179 (91.8)	117 (99.2)	132 (94.3)	
Worse health compared with the past year					
Yes	200 (17.9)	24 (12.3)	12 (10.2)	13 (9.3)	0.006
No	919 (82.1)	171 (87.7)	106 (89.8)	127 (90.7)	
Fever in the past year					
Yes	99 (8.9)	10 (5.1)	10 (8.5)	16 (11.4)	0.211
No	1020 (91.2)	185 (94.9)	108 (91.5)	124 (88.6)	
Asthma attack in the past year					
Yes	44 (3.9)	6 (3.1)	6 (5.1)	9 (6.4)	0.425
No	1075 (96.1)	189 (96.9)	112 (94.9)	131 (93.6)	
Visit mental health professional in the past year					
Yes	65 (5.8)	10 (5.1)	3 (2.5)	9 (6.4)	0.483
No	1054 (94.2)	185 (94.9)	115 (97.5)	131 (93.6)	

*HRQoL* Health-related quality of life

Comparison of medical resource use showed that the proportion of routine place for healthcare visit was similar among the four groups (Table 3). The no exercise group had the highest proportions of emergency, hospitalization, and total healthcare visits: visited ED most often for healthcare (2.1%), had ≥4 healthcare visits/year (73.7%), were hospitalized overnight in the past year (29.3%), and visited ED for asthma in the past year (1.7%).

**Table 3:** Comparison of medical resource use among the four groups by descriptive analyses

Subtype of medical resource use	No exercise n = 1119	Low-intensity exercise n = 195	Moderate-intensity exercise n = 118	High-intensity exercise n = 140	*p*-value
Routine place to go for healthcare					0.998
Yes	1101 (98.4)	192 (98.5)	116 (98.3)	138 (98.6)	
No	18 (1.6)	3 (1.5)	2 (1.7)	2 (1.4)	
Doctor’s office or HMO as most often place for healthcare					0.122
Yes	786 (70.2)	143 (73.3)	94 (79.7)	95 (67.9)	
No	333 (29.8)	52 (26.7)	24 (20.3)	45 (32.1)	
Clinic or health center as most often place for healthcare					0.255
Yes	243 (21.7)	37 (19.0)	18 (15.3)	34 (24.3)	
No	876 (78.3)	158 (81.0)	100 (84.8)	106 (75.7)	
ED as most often place for healthcare					0.794
Yes	23 (2.1)	4 (2.1)	1 (0.9)	2 (1.4)	
No	1096 (97.9)	191 (98.0)	117 (99.2)	138 (98.6)	
Number of healthcare visits/year					0.339
0–3 visits	294 (26.3)	58 (29.7)	38 (32.2)	43 (30.7)	
≥ 4 visits	825 (73.7)	137 (70.3)	80 (67.8)	97 (69.3)	
Hospitalized overnight in the past year					0.008
Yes	328 (29.3)	45 (23.1)	26 (22.0)	25 (17.9)	
No	791 (70.69)	150 (76.92)	92 (77.97)	115 (82.14)	
ED visit for asthma in the past year					0.581
Yes	19 (1.7)	1 (0.5)	1 (0.9)	2 (1.4)	
No	1100 (98.3)	194 (99.5)	117 (99.2)	138 (98.6)	

*HMO* health maintenance organization; *ED* emergency department

Multivariate logistic regression analyses showed that the moderate-intensity exercise group had lower risk for depression over the last 2 weeks than the no exercise group after adjusting for sex, age, race, education, marital status, general health condition, family poverty index ratio, body mass index, cigarette smoking, alcohol drinking, hypertension, cholesterol, chronic heart disease, chest pain, sleep disorders, and cancer (adjusted odds ratio [AOR]: 0.13, 95% CI: 0.02–0.92; Table 4). In the comparison of worse health between the present and past years, the moderate- and high-intensity exercise groups had lower risk than the no exercise group in the crude analysis (odds ratio [OR]: 0.52, 95% CI: 0.28–0.96 and OR: 0.47, 95% CI: 0.26–0.85, respectively). However, the difference lost the significance after adjusting for sex, age, race, education, marital status, general health condition, family poverty index ratio, body mass index, cigarette smoking, alcohol drinking, hypertension, cholesterol, chronic heart disease, chest pain, sleep disorders, and cancer (AOR: 0.83, 95% CI: 0.43–1.58 and AOR: 0.72, 95% CI: 0.38–1.35, respectively). The high-intensity exercise group had higher risk of asthma attack in the past year than the no exercise group (AOR: 2.36, 95% CI: 1.03–5.44). The high-intensity exercise group had a trend for lower risk of overnight hospitalization in the past year than the no exercise group (AOR: 0.65, 95% CI: 0.40–1.04, *p* = 0.075; Table 5).

**Table 4:** Comparison of HRQoL among the four groups by multivariate logistic regression analyses

Subtype of HRQoL	OR (95% CI)	*p*-value	AOR (95% CI)^a^	*p*-value
Depression over the last 2 weeks				
No exercise	1.00		1.00	
Low-intensity exercise	0.74 (0.43–1.28)	0.288	1.02 (0.56–1.84)	0.958
Moderate-intensity exercise	0.07 (0.01–0.51)	0.009	0.13 (0.02–0.92)	0.041
High-intensity exercise	0.51 (0.24–1.06)	0.069	0.70 (0.32–1.55)	0.380
Worse health compared with the past year				
No exercise	1.00		1.00	
Low-intensity exercise	0.65 (0.41–1.02)	0.058	0.87 (0.54–1.41)	0.573
Moderate-intensity exercise	0.52 (0.28–0.96)	0.038	0.83 (0.43–1.58)	0.562
High-intensity exercise	0.47 (0.26–0.85)	0.012	0.72 (0.38–1.35)	0.301
Fever in the past year				
No exercise	1.00		1.00	
Low-intensity exercise	0.56 (0.29–1.09)	0.086	0.55 (0.27–1.10)	0.092
Moderate-intensity exercise	0.95 (0.48–1.88)	0.892	1.17 (0.57–2.40)	0.670
High-intensity exercise	1.33 (0.76–2.33)	0.319	1.57 (0.84–2.91)	0.157
Asthma attack in the past year				
No exercise	1.00		1.00	
Low-intensity exercise	0.78 (0.33–1.85)	0.566	0.86 (0.34–2.14)	0.740
Moderate-intensity exercise	1.31 (0.55–3.14)	0.547	2.27 (0.88–5.83)	0.089
High-intensity exercise	1.68 (0.80–3.52)	0.170	2.36 (1.03–5.44)	0.043
Visit mental health professional in the past year				
No exercise	1.00		1.00	
Low-intensity exercise	0.88 (0.44–1.74)	0.706	0.82 (0.40–1.71)	0.602
Moderate-intensity exercise	0.42 (0.13–1.37)	0.151	0.51 (0.15–1.68)	0.264
High-intensity exercise	1.11 (0.54–2.29)	0.769	0.97 (0.44–2.11)	0.932

*HRQoL* Health-related quality of life; *OR* odds ratio; *AOR* adjusted odds ratio; *CI* confidence interval. ^a^Adjusted for sex, age, race, education, marital status, general health condition, family poverty index ratio, body mass index, cigarette smoking, alcohol drinking, hypertension, cholesterol, chronic heart disease, chest pain, sleep disorders, and cancer

**Table 5:** Comparison of medical resource use among the four groups by multivariate logistic regression analyses

Subtype of medical resource use	OR (95% CI)	*p*-value	AOR (95% CI)^a^	*p*-value
Routine place to go for healthcare				
No exercise	1.00		1.00	
Low-intensity exercise	1.05 (0.31–3.59)	0.943	0.80 (0.21–2.99)	0.737
Moderate-intensity exercise	0.95 (0.22–4.14)	0.944	0.86 (0.18–4.06)	0.846
High-intensity exercise	1.13 (0.26–4.91)	0.873	1.20 (0.25–5.82)	0.822
Doctor’s office or HMO as most often place for healthcare				
No exercise	1.00		1.00	
Low-intensity exercise	1.17 (0.83–1.64)	0.382	1.09 (0.76–1.58)	0.640
Moderate-intensity exercise	1.66 (1.04–2.64)	0.034	1.49 (0.91–2.44)	0.113
High-intensity exercise	0.90 (0.61–1.30)	0.562	0.96 (0.64–1.46)	0.857
Clinic or health center as most often place for healthcare				
No exercise	1.00		1.00	
Low-intensity exercise	0.84 (0.57–1.24)	0.389	0.92 (0.61–1.39)	0.686
Moderate-intensity exercise	0.65 (0.39–1.09)	0.105	0.73 (0.42–1.25)	0.250
High-intensity exercise	1.16 (0.77–1.75)	0.489	1.10 (0.70–1.73)	0.682
ED as most often place for healthcare				
No exercise	1.00		1.00	
Low-intensity exercise	1.00 (0.34–2.92)	0.997	1.17 (0.37–3.68)	0.791
Moderate-intensity exercise	0.41 (0.16–3.04)	0.382	0.52 (0.07–4.11)	0.533
High-intensity exercise	0.69 (0.16–2.96)	0.618	0.81 (0.17–3.85)	0.786
Healthcare visits ≥ 4/year				
No exercise	1.00		1.00	
Low-intensity exercise	0.84 (0.60–1.18)	0.313	0.91 (0.64–1.30)	0.601
Moderate-intensity exercise	0.75 (0.50–1.13)	0.168	0.89 (0.58–1.37)	0.595
High-intensity exercise	0.80 (0.55–1.18)	0.264	1.01 (0.67–1.53)	0.948
Hospitalized overnight in the past year				
No exercise	1.00		1.00	
Low-intensity exercise	0.72 (0.51–1.03)	0.076	0.88 (0.61–1.29)	0.518
Moderate-intensity exercise	0.68 (0.43–1.07)	0.098	0.83 (0.52–1.34)	0.453
High-intensity exercise	0.52 (0.33–0.82)	0.005	0.65 (0.40–1.04)	0.075
ED visit for asthma past year				
No exercise	1.00		1.00	
Low-intensity exercise	0.30 (0.04–2.24)	0.240	0.31 (0.04–2.45)	0.266
Moderate-intensity exercise	0.50 (0.07–3.73)	0.495	0.95 (0.12–7.66)	0.958
High-intensity exercise	0.84 (0.19–3.64)	0.815	0.92 (0.18–4.64)	0.918

*OR* odds ratio; *AOR* adjusted odds ratio; *CI* confidence interval; *HMO* health maintenance organization; *ED* emergency department. ^a^Adjusted for sex, age, race, education, marital status, general health condition, family poverty index ratio, body mass index, cigarette smoking, alcohol drinking, hypertension, cholesterol, chronic heart disease, chest pain, sleep disorders, and cancer

## Discussion

The present study showed that male participants with higher education performed more exercise than their counterparts. Participants with moderate- and high-intensity exercise self-reported better general health condition than participants without exercise. Participants without exercise had the highest proportions of being overweight and cigarette smoking. Compared with exercise groups, the number of participants who reported depression over the last 2 weeks and worse health compared with the past year was higher in the no exercise group. In the comparison of medical resource use, participants without exercise had the highest proportions of emergency, hospitalization, and total healthcare visits: visited ED most often for healthcare, had ≥4 healthcare visits/year, were hospitalized overnight in the past year, and visited ED for asthma in the past year. Multivariate logistic regression analyses showed that the moderate-intensity exercise group had significantly lower risk of depression over the last 2 weeks than the no exercise group. The high-intensity exercise group had significantly higher risk of asthma attack in the past year than the no exercise group. Other differences among the four groups were not significant.

The improvements of HRQoL including lower depression in participants with moderate-intensity exercise may be due to better glycemic control, increased muscle strength, increased power and functional capacity, and better cardiovascular function [4]. The mechanisms of better glycemic control by exercise include increases in insulin sensitivity, glucose transporter protein-4 translocation to the muscle cell membrane, glycogen synthase activity, glucose metabolism by increased muscle mass [10, 11], available glucose storage capacity, and glucose clearance from the circulation and reduction in visceral fat [11]. Aerobic and resistance trainings effectively reduced the glycemic levels in individuals with diabetes and even prediabetes [11–13]. A previous work suggested effective aerobic training including continuous low to moderate-intensity or intermittent high-intensity training 3–5 times per week [4]. Resistance training combining heavy and explosive loads could improve insulin sensitivity and decrease abdominal fat in elderly people with diabetes [11]. The combination of aerobic and resistance trainings is more effective to improve neuromuscular and cardiovascular functions than aerobic or endurance training alone [4]. The level of exercise intensity is also important. Exercise is suggested to be composed of at least 150 min of exercise per week, and more exercise is considered be better, divided into 2 or 3 nonconsecutive days [12]. This study showed that participants with high-intensity exercise had lower depression than those without exercise; however, the difference was not significant. The possible explanation is the limited sample size. We suggest larger studies about this issue in the future.

Elderly people with diabetes have higher risk of depression than those without diabetes [14]. People with depression also have an increased risk of diabetes [15]. Depression contributes to poor compliance of diabetes management, provider–patient communication, and therapeutic effects [16]. In addition, a recent study reported that depression may accelerate cognitive decline in people with diabetes [16]. A systemic review reported that exercise appears to exert beneficial clinical effects on elderly people with depressive symptoms [17]. The guideline of National Institute for Health and Clinical Excellence also suggested structured and supervised exercise programs for people with mild to moderate depression [18].

This study showed that participants without exercise had higher proportions of ED visit, total healthcare visit/year, and overnight hospitalization than the three exercise groups. These findings suggest that lack of exercise may predispose elderly people with diabetes to higher risk for frailty and subsequent complications [4]. The risk of asthma attack in the past year was higher in the high-intensity exercise group than in the no exercise group. Although exercise is a risk factor for asthma attack [19], respiratory exercise program was found to increase muscle strength, patient health, and quality of life [20]. Male participants with higher education performed more exercise, consistent with previous reports [21–23]. The positive association between higher education and more exercise suggests that education for exercise and its related benefits is important for promoting health in elderly people with diabetes.

The strength of this study is that it clarified an unclear issue by using population-based data. The limitations are as follows. First, the study adopted a cross-sectional design; as such, we could only figure out that exercise was associated with HRQoL, but the causal relationship could not be inferred. Second, the exercise level, comorbidities, HRQoL, and medical resource use were self-reported and therefore may have subjective bias. Third, the number of participants in the three exercise groups was relatively small. A higher number of participants is warranted for further investigation. Fourth, the result may not be generalized to other nations due to differences in culture, race, and medical resource.

## Conclusion

This cross-sectional population-based study showed that exercise was associated with better HRQoL and lack of exercise was associated with higher medical resource use in elderly people with diabetes. Exercises including aerobic and resistance training are suggested for elderly people with diabetes. However, further studies including cohort design and more participants must be conducted to validate the results.

## Data Availability

The datasets generated and/or analyzed during the current study are available in the NHANES.

## Abbreviations

HRQoL: Health-related quality of life
NHANES: National Health and Nutrition Examination Survey
ED: Emergency department
AOR: Adjusted odds ratio
CI: Confidence interval
OR: Odds ratio
